# Suboptimal compliance with surgical safety checklists in Colorado: A prospective observational study reveals differences between surgical specialties

**DOI:** 10.1186/s13037-014-0056-z

**Published:** 2015-01-31

**Authors:** Walter L Biffl, Annalee W Gallagher, Fredric M Pieracci, Crystal Berumen

**Affiliations:** Department of Surgery, Denver Health Medical Center, 777 Bannock St., MC 0206, Denver, CO 80204 USA

**Keywords:** Checklist, Surgery, Safety, Compliance, Observation, Implementation, Surgeons, Operating room, Sentinel events

## Abstract

**Background:**

Surgical safety checklists (SSCs) are designed to improve team communication and consistency in care, ultimately avoiding complications. In Colorado, hospitals reported that use of SSCs was standard practice, but a statewide survey indicated that SSC use was inconsistent. The purpose of this project was to directly observe the compliance with the SSC in Colorado hospitals, through direct observation of the perioperative checklist process.

**Methods:**

Ten hospitals participated in a quality improvement initiative. Trained team members recorded compliance with each of the components of the SSC. Data analysis was performed using a chi-squared test or ANOVA, depending on the number of categorical variables, with p < 0.05 determining statistical significance.

**Results:**

Ten hospitals representing statewide diversity submitted 854 observations (median 98, range 24–106). 83% of cases were elective, 13% urgent, and 4% emergent/trauma. There was significant variation across hospitals in: team introductions, cessation of activity, affirming correct procedure, assessing hypothermia risk, need for beta blocker, or VTE prophylaxis. Uniformly poor compliance was observed with respect to assessment of case duration, blood loss, anesthesiologists’ concerns, or display of essential imaging. Only 71% of observers reported active participation by physicians; 9% reported that “the majority did not pay attention” and 4% reported that the team was “just going through the motions”. There were significant differences among surgical specialty groups in the majority of the elements.

**Conclusion:**

SSCs have been implemented by the vast majority of hospitals in our state; however, compliance with SSC completion in the operating room has wide variation and is generally suboptimal. Although this study was not designed to correlate SSC compliance with outcomes, there are concerns about the risk of a sentinel event or unanticipated complication resulting from poor preparation.

## Background

In 2009, The New England Journal of Medicine published a special article entitled, *A Surgical Safety Checklist to Reduce Morbidity and Mortality in a Global Population* [1]. This study, supported by the World Health Organization (WHO), described the use of a 19-item surgical safety checklist (SSC) that was designed to improve team communication and consistency in care in the perioperative period. After implementation of the WHO SSC in eight diverse institutions around the globe, there were statistically significant reductions in the rates of death and complications [1]. Following this publication, use of checklists was broadly embraced and strongly encouraged by groups such as WHO and the Institute for Healthcare Improvement, to the point that use of SSCs was mandated in the UK and Canada [2-4]. In the United States, many hospitals implemented surgical checklists, often adapting the original WHO SSC to fit individual facility needs [5,6]. Momentum behind checklist initiatives has grown as accumulating literature has suggested that implementation of SSCs is associated with reductions in postoperative complications and mortality [7-10].

**Figure 1:**
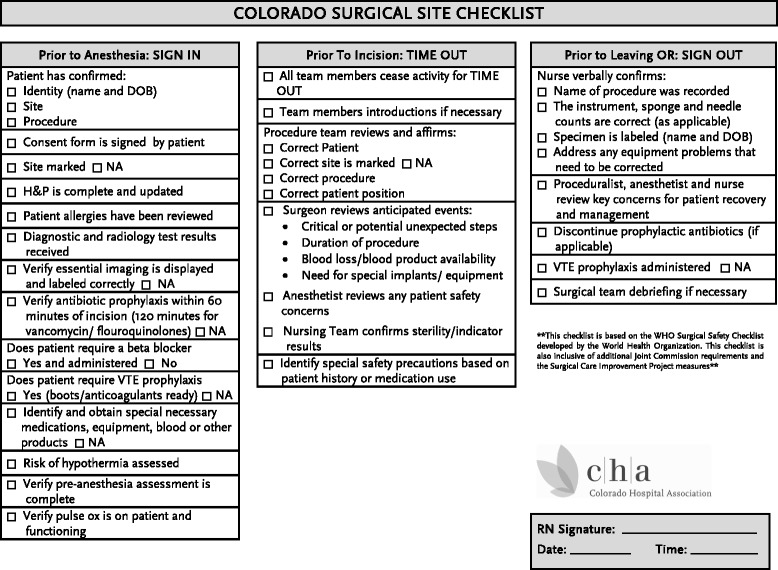
**The Colorado hospital association surgical safety checklist.** Based on the World Health Organization checklist, this incorporated Surgical Care Imptrovement Project initiatives.

In Colorado in 2010, the Colorado Hospital Association (CHA) partnered with the Colorado Medical Society and COPIC- the major medical liability carrier for physicians and hospitals in Colorado- to modify and standardize the WHO checklist to include other Joint Commission requirements as well as Surgical Care Improvement Project (SCIP) measures (Figure 1). In a subsequent CHA survey, over 90% of responding hospitals reported implementation of the “Colorado SSC” in their surgical areas (CHA, unpublished data). However, CHA and COPIC were concerned that the failure to consistently review all of the checklist items might put patients at risk of “Never Events” such as wrong-patient/wrong-site procedures or retained foreign bodies [11]. In fact, in spite of the promulgation of National Patient Safety Goals, these events had continued to occur in Colorado [12]. The purpose of this quality improvement project was to observe the level of compliance with discrete components of the SSC in Colorado hospitals, through direct observation of the perioperative checklist process.

## Methods

This observational quality improvement project was carried out between September 2012 and April 2013 at ten selected Colorado hospitals. As a quality improvement project, it was exempt from Institutional Review Board approval, and individual patients’ protected health information was never collected or transmitted. The hospitals were selected by COPIC and CHA with the goal of ensuring a representative sample of urban vs rural and academic vs community hospitals; in addition, some hospitals were primarily insured by COPIC, and some were not. All facilities that were invited to participate agreed to do so, and all ten completed the project.

**Figure 2:**
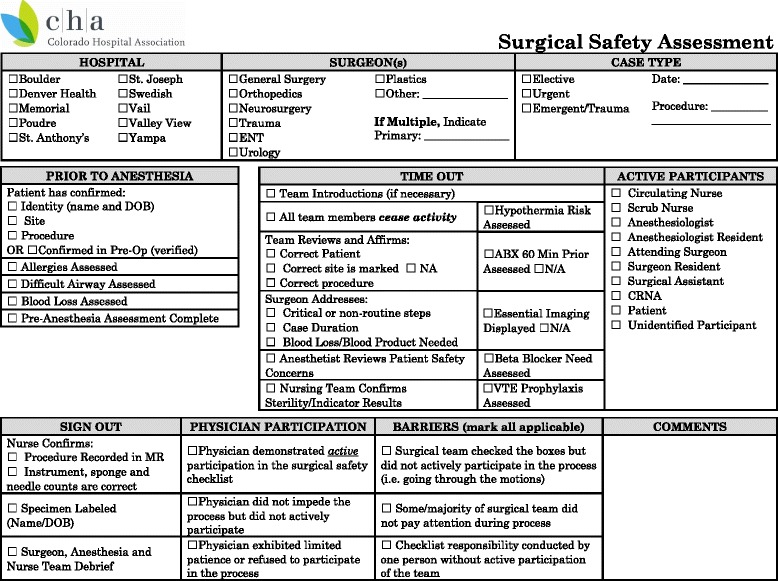
**The surgical safety assessment form.** This observation tool was based on the Colorado Hospital Association Surgical Safety Checklist. It was modified to group items according to the perioperative workflow, for ease of completion during the process.

A preliminary meeting was held in which representatives from CHA and the ten hospitals discussed the hospitals’ current surgical workflow and checklist use. There was no implementation intervention performed; this project was intended to assess existing levels of compliance. It was agreed that, for the purposes of the project, the CHA SSC elements would be used as the basis for comparison. A standardized assessment tool- the CHA “Surgical Safety Assessment (SSA)” form (Figure 2)- was created for the observations. The SSA form was designed to reflect the typical surgical workflow, and thus varied slightly from the CHA SSC form. For example, the display of essential imaging and choice of appropriate venous thromboembolism (VTE) prophylaxis is generally the responsibility of the surgeon, so these observations were grouped in the “Time Out” section, when the surgeon would be present, rather than the “Prior to Anesthesia” section, when the surgeon is not typically present.

Once the SSA form was completed and distributed, a final workshop was held in which the observers from each site were trained to use the SSA form and to perform observations as discreetly as possible, without the knowledge of the operating team. The observer was generally a member of the operating room (OR) staff, whose presence would not raise suspicion, but who was not directly involved in that procedure. In addition to review of the safety elements, the observers recorded team participation, the role and participation of physicians, and what they perceived as barriers to consistent use. The completed assessments, which contained no protected health information or other patient-identifying information, were submitted to CHA, where information from the SSA forms was entered into a database.

Each hospital was asked to perform 100 observations. This was intended to avoid having the sample size dominated by the busiest hospitals. The hospitals were also requested to perform observations in a case mix that approximated their overall case mix (eg, urgent: elective cases, and across specialties in a representative approximation).

### Statistical analysis

All statistical analyses were performed using SAS version 9.2 (SAS Inc., Carey, NC, USA). Data are expressed as number (percentage). All outcomes were dichotomous categorical. Cell with either missing data or “non-applicable” values were omitted from the analysis; as such the total sample size for each independent variable analyzed differed. Analysis of independent variables was first performed across all observations and then among the following subgroups: 1) by hospital, 2) by specialty (general surgery, orthopedic surgery, neurosurgery, and other), and 3) by hospital infection rate (high vs. low, dichotomized around the median infection rate of 0.5%). Differences in categorical variables between two groups were assessed using the chi-squared test, unless expected cell counts were less than 10, in which case Fischer’s exact test was used. Differences in categorical variables between more than two groups (e.g., specialty) were assessed using ANOVA. The alpha error level was set at 0.05, with p < 0.05 being considered statistically significant.

## Results

**Figure 3:**
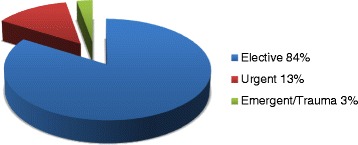
**Distribution of case type (n = 854).**

**Figure 4:**
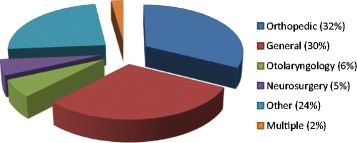
**Distribution of surgical specialty type (n = 854).**

The ten participating hospitals were diverse. Three were rural and seven were urban/suburban. Two of the ten hospitals were academic (ie, staffed with surgical residents), and the other eight were community hospitals. Five were designated as level I or II trauma centers by the State of Colorado, and five were not. The original goal was 100 observations per site. A total of 854 observations were made (per-hospital median 98, range 24–106). All hospitals completed their observations within a 6-week time frame. Overall, 83% of cases were elective, 13% were urgent, and 4% emergent/trauma (Figure 3). The majority of cases were performed by orthopedic (32%) and general (30%) surgeons. Otolaryngologists performed 6% and neurosurgeons 5%; 24% were done by others (obstetrician/gynecologists, oral/maxillofacial surgeons, plastic surgeons, etc.), and 2% of cases had two or more surgical specialties involved (Figure 4).

**Figure 5:**
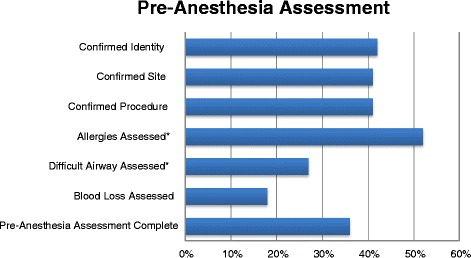
**Compliance with pre-anesthesia assessment checklist components prior to induction of anesthesia.** * = significant variation across hospitals, p < 0.05.

The SSA contains elements pertaining to a pre-anesthetic timeout; compliance with these elements is listed in Figure 5. There were three hospitals in which this was completed ≤5% of the time, while in two hospitals, at least some elements were reviewed >90% of the time. Overall, the components of this segment of the SSA were reviewed in fewer than 50% of patients.

**Figure 6:**
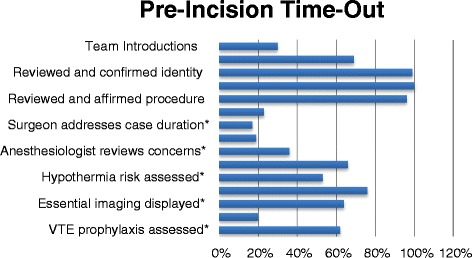
**Compliance with pre-incision time-out checklist components prior to initiation of surgical procedure.** * = significant variation across hospitals, p < 0.05.

**Figure 7:**
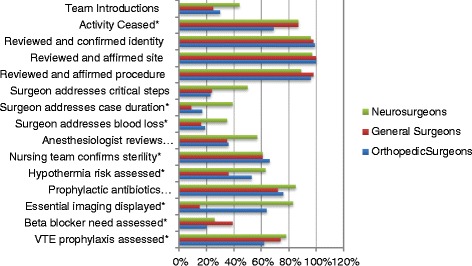
**Compliance with checklist components prior to initiation of surgical procedure, by procedure.** * = significant variation across specialty groups, p < 0.05.

Compliance with the pre-incision segment of the SSA was better in several elements, but not all (Figure 6). In nearly all (95-99%) cases the correct patient and procedure were verified, and the site was confirmed in 91% of relevant cases. There was significant hospital-to-hospital variation in addressing the following (overall mean in parentheses): team introductions (30%), cessation of activity (77%), assessing hypothermia risk (48%), need for beta blocker (23%), and VTE prophylaxis (65%). Uniformly poor compliance, without significant variation, was observed with respect to assessment of case duration (16%), blood loss (19%), anesthesiologists’ concerns (39%), and display of essential imaging (36%). Interestingly but perhaps not surprisingly, there were significant differences among surgical specialty groups in the majority of the elements (Figure 7). Compared with orthopedic and neurosurgeons, general surgeons were less compliant with team introductions, addressing critical steps, case duration, blood loss, anesthesia concerns, hypothermia risk, antibiotic administration, or displaying imaging. Orthopedic surgeons were less compliant with ceasing activity, and with assessing the need for beta-blockers or VTE prophylaxis.

Only 71% of observers perceived active participation by physicians; 9% reported that “the majority did not pay attention” and 4% reported that the team was “just going through the motions.” Compared with orthopedic and neurosurgeons, general surgeons were more compliant with active participation.

## Discussion

In this observational study, we have found that although over 90% of Colorado hospitals reported utilizing checklists in the OR, compliance with the Colorado SSC is consistently inconsistent and incomplete. This is not unique to Colorado. Pickering and colleagues [3] observed 294 operations performed over five different hospitals in the United Kingdom, and reported results similar to ours: although administrative audits indicated use of checklists in 95% of cases, active participation was observed in only 73%, and all information was communicated in just 55% of cases [3]. In the Netherlands, where checklist use was mandated by the Dutch Health Care Inspectorate, the checklist was fully completed in just 39% of cases [7]. In fact, as more studies have emerged, it has become clear that checklist implementation does not equate with compliance [8]. In one prospective observational study, Levy and colleagues [13] found that, despite 100% documented completion of the preincision phase of the checklist, most of the individual elements were either not addressed as designed, or not addressed at all. Similarly, Sparks and colleagues [14] found that in spite of a high level of overall participation and completion, the accuracy was poor.

The finding of poor overall compliance was not surprising- when Colorado hospitals had initially been surveyed about checklist use by CHA, one-third had raised concerns over the degree of active physician participation, and many suggested that the checklist is not consistently used on every surgical case. The need for active participation and a cultural change is a recurring theme in checklist implementation literature [8,15,16]. O’Connor and colleagues [17] examined the “human factors” in interviews with operating team members, and identified critical factors to improvement: 1) involvement of all the operating team members in the checklist process; 2) support from senior personnel; 3) ongoing education and training; and 4) breakdown of barriers to implementation. This project neither prescribed nor assessed implementation strategies. However, the barriers reported by the observers in the present study were consistent with these concepts.

In the present study, compliance varied across hospitals and by elements of the checklist. Variation across hospitals has been noted in other studies [3,18-20]. For example, van Schoten and colleagues [19] found that compliance in the Netherlands was inferior at academic hospitals, compared with general hospitals and teaching hospitals. They point out that the literature is mixed on whether large or small hospitals perform better [19]. In the present study, there was no statistical difference in compliance between the five highest-volume hospitals and the five lower-volume hospitals, although th e dichotomy was not substantial. This may merit further study.

The pre-anesthesia timeout was performed sporadically in the OR. This may have been due to the common practice of anesthesiologists to see patients in the preoperative holding area just moments before the patient being transported into the OR. Other elements of the pre-anesthesia assessment might be performed by the anesthesiologist but not reviewed with the nurses. In addition, the surgeon may not be present in the OR prior to induction of anesthesia, so elements might be “saved” for discussion with the surgeon. This is something to be explored in the future.

The pre-incision timeout did not have as much variation as the pre-anesthetic pause. Indeed, the culture of confirming patient/procedure/site seems to be well-ingrained. In studies directly assessing compliance, these items are consistently addressed [13,18]. On the other hand, elements that individually seem important are not routinely reviewed. From a surgeon’s perspective, this is understandable to a degree. Team introductions are not necessary if the team works together frequently; this may not have been noted in the observations. “Critical steps” may not seem necessary to review in “routine” procedures such as cholecystectomy or hernia repair. Similarly, anesthesiologists’ concerns, anticipated blood loss, case duration, display of imaging, or hypothermia may not seem relevant to brief, common procedures. However, it is important to remember that the checklist concept is designed to make a habit of getting all of the team members to speak up, and to discuss every item as they are individually important to surgical planning.

A noteworthy, but probably not surprising, finding of this study was that compliance with individual elements of the SSC varied by surgical specialty. This has been reported by others as well [18,19,21]. In the present study, general surgeons were less compliant with team introductions, addressing critical steps, case duration, blood loss, anesthesia concerns, hypothermia risk, antibiotic administration, or displaying imaging. There may be a variety of explanations for this. Teams that work together frequently may not feel compelled to introduce themselves; the observer may not have noted previous familiarity. We did not record details of operative procedures, so it is difficult to know whether critical steps, blood loss, hypothermia risk, or imaging were relevant- if the majority of cases were routine procedures (eg, hernia repairs, cholecystectomies) then it could be argued that such points were not germaine. On the other hand, it is not known whether unexpected occurrences arose during any of these procedures, and how they were handled. Orthopedic surgeons were less compliant with ceasing activity, and with assessing the need for beta-blockers or VTE prophylaxis. Without outcomes data (wrong site procedures, perioperative myocardial infarctions, venous thromboembolic events, etc.) it is not known whether or not these failures are problematic. The fact that general surgeons appear less compliant with certain measures may simply be a reflection of case complexity. Of note, although there is variability by surgical specialty, the “nurse-driven” components of the SSC (e.g. patient and site identity, and verification of instrument sterility) did not show any variation across specialties. This suggests that the nurses are consistent in initiating the SSC review, but that review of many individual elements is left to the surgeon. Interestingly, although the general surgeons were inferior in reviewing many checklist elements, the observers felt that the general surgeons were more compliant with active participation overall. This may indicate an opportunity for teamwork training with certain specialties/service lines.

The present study did not assess outcomes, but it is assumed that poor compliance puts patients at risk. The question is, what risk? Haynes and colleagues [1] reported that death and postoperative complications improved following implementation of SSCs. A before-and-after cohort study in the Netherlands similarly found that, after adjusting for case mix, implementation of checklists was associated with a significant reduction in mortality [7]. A subsequent review and meta-analysis- which included both the Haynes [1] and van Klei [7] studies- concluded that the evidence is “highly suggestive of a reduction in postoperative complications and mortality following implementation of the WHO SSC”- but the authors concluded that the evidence could not be regarded as definitive in the absence of higher-quality studies [9]. Indeed, a close look at the report of Haynes et al. [1] reveals that five of the eight hospitals had no significant improvement in the measured outcomes, and that baseline death and complication rates were higher than what is reported in US hospitals. And recently, Urbach and colleagues [4] reported that large community hospitals in Ontario nearly all reported checklist compliance in 99-100% of cases, and yet there was no reduction in operative mortality or complications. It was speculated by the authors- [4] and by Leape [22] in an accompanying editorial- that the failure to improve outcomes may have been related to poor compliance with checklist elements. However, without a clear link between compliance and outcomes in controlled studies, it is just as likely that the “positive” studies (e.g., Haynes et al., [1] van Klei et al. [7]) were confounded by the Hawthorne effect or other unknown factors.

One might argue that it is unrealistic to expect that completing a checklist in the OR will prevent mortality or major surgical complications. The existing literature does not indicate how often the checklist process identifies or averts an error (“near-misses”) or enhances preparation for a difficult case. On the other hand, there are some outcomes that absolutely should improve or be prevented altogether by reviewing a checklist. Appropriate verification of patient, procedure and site should completely eliminate wrong patient/wrong site procedures. Review of all SSC elements should also ensure compliance with core measures, which has very real financial repercussions for hospitals. In addition, a brief review of expected procedural steps and special equipment needs should improve OR efficiency by having necessary equipment readied in advance. Further, discussion of anticipated critical steps and blood loss may enhance the team’s preparation for dealing with challenging intraoperative events. Finally, the open discourse is recognized to improve the perception of teamwork.

A major strength of this study is that direct observations were performed surreptitiously by known members of the operating room staff, thus minimizing the Hawthorne effect. And recording of compliance with all the checklist elements is far more accurate than a simple “yes/no” recording of checklist use, or using surrogates such as core measures data. The recording of additional notations of perceived participation and teamwork, as well as barriers, has allowed us to provide feedback to hospitals that they may use to further educate their staff and improve compliance in the future. Another factor that minimized potential Hawthorne effect was that there was no formal implementation period. Hospitals were assessed in their current state, so there was no “new change” that might have waned over time.

A limitation of the current study is that the number of urgent and emergent cases was relatively low, precluding a separate analysis of those cases. It is generally assumed that compliance is lower in such situations, so team awareness of such challenges is important. In fact, van Klei and colleagues [7] noted that compliance was poor in urgent/emergent cases and cases involving sicker patients at higher risk of dying- and there was no improvement in mortality in these cases. In the Safe Surgery Saves Lives program, although compliance was not perfect, the investigators found a significant improvement in checklist compliance in urgent cases, and a significant reduction in complications after urgent surgery [23]. Another limitation is that details of case complexity were not recorded, precluding our ability to address our hypothesis that many elements were skipped on the basis of it being a “routine” case.

Surgical outcomes were not specifically assessed in this project. Prior to 2010, COPIC collected data on sentinel events; since then, however, only claims data are available- so it is not clear whether wrong patient/wrong site/wrong procedure events were different. In addition, it was impossible to link procedures to postoperative complications or mortality due to de-identification of data. Even if it had been available, the study was not designed to detect mortality differences and was likely underpowered to do so in the routine surgical population.

In sum, although most facilities report adoption and implementation of a comprehensive checklist, consistent adherence and multi-disciplinary participation with respect to the surgical checklist remains suboptimal. These comprehensive lists are inclusive of elements such as appropriate prophylactic antibiotic use prior to incision to lessen the likelihood of surgical-associated infections and appropriate surgical site marking – yet surgical site infections and wrong site procedures continue to be a prevalent problem in Colorado facilities. Surgical site infection (SSI) rates in Colorado facilities have remained relatively similar in the past two years and individual facility rates have largely not made statistical improvements (i.e. moving from a statistically same national comparison to a statistically better national comparison). Clearly, evaluation of implementation, consistent use, compliance and barriers are necessary elements to further study the SSC in Colorado hospitals.

## Conclusions

In conclusion, SSCs have been implemented by the vast majority of hospitals in our state; however, compliance with SSC completion in the OR has wide variation and is generally suboptimal. Although this study was not designate to correlate SSC compliance with outcomes, there are concerns about the risk of a sentinel event or unanticipated complication resulting from poor preparation. There are many intuitively clear benefits to the use of SSCs, including: the absolute prevention of wrong patient/wrong site surgery; compliance with Joint Commission standards and National Hospital Inpatient Quality Measures; and improvement of efficiency by ensuring availability of important equipment and anticipation of contingencies. These outcomes are less easily measured, yet every failure is ultimately costly. Use of SSCs is important, and we feel that the focus should be on supporting local implementation efforts. A second phase of this project is under development to determine how compliance can be improved.

## Abbreviations

WHO: World health organization
SSC: Surgical safety checklist
CHA: Colorado hospital association
SCIP: Surgical care improvement project
SSA: Surgical safety assessment
VTEm: Venous thromboembolism
OR: Operating room
